# Inhibition of miR-497 Attenuates Oral Submucous Fibrosis by Inhibiting Myofibroblast Transdifferentiation in Buccal Mucosal Fibroblasts

**DOI:** 10.3290/j.ohpd.b3276183

**Published:** 2022-08-03

**Authors:** Jie Huang, Huang Zhang, Xusheng Fan, Junxin Guo

**Affiliations:** a Attending Physician, Department of Stomatology, Hangzhou First People’s Hospital Affiliated to Medical College of Zhejiang University, Hangzhou, China. Wrote the manuscript.; b Attending Physician, Department of Stomatology, Hangzhou First People’s Hospital Affiliated to Medical College of Zhejiang University, Hangzhou, China. Performed the assays.; c Attending Physician, Department of Stomatology, Hangzhou First People’s Hospital Affiliated to Medical College of Zhejiang University, Hangzhou, China. Ran the cell cultures.; d Attending Physician, Department of Stomatology, Shulan (Hangzhou) Hospital, Hangzhou, China. Study idea.

**Keywords:** buccal mucosal fibroblasts, miR-497, myofibroblast transdifferentiation, oral submucous fibrosis, TGF-β1/Smads signaling pathway

## Abstract

**Purpose:**

Oral submucous fibrosis (OSF) is a common chronic condition with poor prognosis, and existing therapies for OSF are limited in effectiveness. This study was designed to explore the role of miR-497 in arecoline (AR)-induced OSF.

**Materials and Methods:**

After miR-497 was silenced or overexpressed in buccal mucosa fibroblasts (BMFs), different concentrations of AR (5–200 µg/ml) were applied to incubate BMFs, and 50 µg/ml of AR was chosen for subsequent experiments. Thereafter, collagen gel contraction assay was used to detect the contractile capacity of BMFs. Transwell assay and wound healing assay were applied to detect migration and invasiveness of the cells. In addition, immunofluorescence staining, qRT-PCR and western blot were conducted to measure the expression of miR-497, collagen I and α-SMA, as well as the phosphorylation of Smad2 and Smad3.

**Results:**

After successful inhibition or overexpression of miR-497 in AR-induced BMFs, the results showed that miR-497 inhibition suppressed the contractility, migration and invasiveness of AR-induced BMFs, whereas overexpression of miR-497 produced the opposite. In addition, miR-497 inhibition down-regulated the expression level of collagen I and α-SMA in AR-exposed BMFs. Furthermore, TGF-β1 expression, Smad2 and Smad3 phosphorylation were also repressed in AR-induced BMFs after miR-497 inhibition. Correspondingly, overexpression of miR-497 reversed the expression of the aforementioned proteins.

**Conclusion:**

miR-497 inhibition may attenuate OSF by inhibiting myofibroblast transdifferentiation in BMFs via the TGF-β1/Smads signaling pathway, indicating that miR-497 might represent an underlying target for treating OSF.

Oral submucous fibrosis (OSF) is a chronic disorder, affecting the entire oral cavity and even the esophageal mucosa or pharynx.^9^ OSF patients frequently suffer from limited mouth opening, which critically affects their quality of life due to the adverse effects on chewing, social activities, and oral hygiene.^29^ Despite the availabiltiy of some methods of treating OSF, such as surgery and chemotherapy, the therapeutic efficacy is still limited and the prognosis of OSF patients remains poor.^35^ About 7%-30% of OSF patients will develop into malignancy.^34^ Hence, the development of novel and effective treatments for OSF is critically needed.

The pathogenesis of OSF is complex and involves numerous factors, such as epithelial-mesenchymal transition, extracellular matrix remodeling as well as fibroblast changes.^32^ Myofibroblasts are critical in the process of tissue remodelling and wound healing, but the sustained activation of myofibroblasts frequently results in pathological fibrosis.^38^ Accumulating evidence suggests that myofibroblast transdifferentiation in buccal mucosa fibroblasts (BMFs) plays a key role in OSF pathogenesis.^27^ Fibrosis of BMFs will lead to hypovascularity and ensuing oral mucosa blanching, gingival and tooth staining and trismus.^27^ In OSF patients, increased fibrosis severity always portends poorer outcome.^18,28^ Therefore, approaches that reduce myofibroblast transdifferentiation in BMFs may be beneficial for treating OSF.

MicroRNAs (miRNAs) are a category of single-stranded, non-coding, short RNAs, which modulate protein-coding gene expression at a post-transcriptional level.^12^ Accumulating evidence shows that miRNAs are important in ameliorating fibrotic diseases by inhibiting fibroblast-to-myofibroblast transdifferentiation, such as OSF.^13,21^ A study conducted by Chen et al^5^ has found that miRNA-497 expression profiles are changed in a model of idiopathic pulmonary fibrosis; upregulating the expression of miR-497-5p can induce myofibroblast transdifferentiation in lung resident mesenchymal stem cells and promote pulmonary fibrogenesis. Nevertheless, the regulation of myofibroblast transdifferentiation by miR-497 in OSF has to date been seldom reported.

Hence, this study aimed to evaluate the effect and detailed mechanism of miR-497 in arecoline (AR)-induced OSF in BMFs. Specifically, after measuring miR-497 expression in BMFs, the effects of miR-497 inhibition or overexpression in AR-induced myofibroblast transdifferentiation in BMFs were investigated, including cell contractility, migration and invasion abilities. In addition, the expression of fibrosis-related factors and the relationship between miR-497 and the TGF-β1/Smads signaling pathway in BMFs were also evaluated, which providing a novel scientific basis for the treatment of OSF.

## Materials and Methods

### Cell Culture and Grouping

BMFs were obtained from iCell Bioscience (Shanghai, China). After washing twice with PBS buffer containing antibiotics (100 U/ml penicillin, 100 μg/ml streptomycin and 0.25 μg/ml of fungizone), the BMFs were cultured in low glucose DMEM with 10% fetal bovine serum (FBS, Hyclone; Logan, UT, USA) and antibiotics as described above. The cells at passages 3-8 were selected for subsequent experiments.

For transdifferentiation induction, the medium was replaced by fresh serum-free medium and cultured for 18 h, followed by incubation in medium containing AR (50 μg/ml) for 24 h. The BMFs treated identically but without AR comprised the normal control group (NC). The AR-stimulated BMFs were divided into 5 groups: AR group, miR-497 silence group (AR+sh-miR-497), miR-497 silence vector group (AR+sh-miR-NC), miR-497 overexpression group (AR+OE-miR-497) and miR-497 overexpression vector group (AR+OE-NC).

### Lentiviral-mediated Silencing of miR-497

The double-stranded short hairpin RNA (shRNA) sequence was cloned in strict accordance with the instruction leaflet. A shRNA specifically targeting human miR-497 was constructed and cloned into the pLV-RNAi to produce lentiviral vectors.

### Overexpression of miR-497

cDNA of miR-497 was cloned in pLV-EF1a-MCS-IRES-Puro. To produce the lentivirus, 293T cells were cotransfected with plasmid DNA mixture and helper plasmids by Lipofectamine 2000 (LF2000, Invitrogen; Carlsbad, CA, USA).

### qRT-PCR

Total RNA from the BMFs was isolated with RNA extraction reagents (Sangon Biotech; Shanghai, China). Subsequently, the extracted RNA was reverse-transcribed into cDNA by RNA reverse-transcription kit (CWBIO; Beijing, China) based on the manufacturer’s instructions. After that, qRT-PCR was performed with SYBR Premix Ex TaqII (Takara; Kusatsu, Japan) to determine the relative mRNA expression levels. GAPDH served as an internal reference. The sequences of the qRT-PCR primers used in this study were included in Table 1.

**Table 1:** qRT-PCR primers

Gene	Forward primer	Reverse primer
Human miR-497	AGTCCAGTTTTCCCAGGAATCCCT	ACCAGCAGCACACTGTGGTTTGT
Human α-SMA	CCCAGATTATGTTTGAGACCTTC	ATCTCCAGAGTCCAGCACAATAC
Human Collagen I	CTTCTGGTCCTCGTGGTCTCCCT	AAGCCTCGGTGTCCCTTCATTCC
Human TGF-β1	AGACTTTTCCCCAGACCTCG	GGGGTGTCTCAGTATCCCAC
Human GAPDH	CGGTGCTGAGTATGTCGTGGAGT	ACAGTCTTCTGGGTGGCAGTGAT

### Collagen Gel Contraction Assay

The cells were resuspended at a concentration of 2 x 10^5^/ml in 2 mg/ml collagen solution. The cell-collagen mixture was seeded in 24-well plates (0.5 ml/well) and incubated for collagen gel polymerization. Afterwards, the gels were removed from the wells with a sterile pipette tip. The relative gel area was calculated by Image J software (NIH; Bethesda, MD, USA) and determined as the percentage of the final gel area compared to the original gel area.

### Transwell Assay

Cells of the six groups were collected and prepared as cell suspensions. For migration assay, cell suspension (200 µl) without FBS was injected into the upper chamber, while the lower chamber was loaded with high-glucose DMEM containing 10% FBS. Following incubation at room temperature for 24 h, the cells at the bottom of the upper chamber were wiped off with cotton-tipped swabs. The BMFs that had migrated into the lower chamber were fixed using 4% paraformaldehyde (Maklin; Shanghai, China), stained using 0.1% crystal violet (Shanghai Qiangshun Chemical Reagents; Shanghai, China) for 30 min and counted by light microscope. For the cell invasion assay, the upper chamber of the Transwell was coated with 30 μl Matrigel (Millipore; Bedford, MA, USA), and the other steps were similar to those of the migration assay.

### Wound Healing Assay

Before performing the wound healing assay, a marking pen was used to draw horizontal lines (every 1 cm across the hole and 5 lines per hole) on the back of the 6-well plate. Then, the cells were plated in each well, and each plate was placed in the cell incubator overnight. When confluent, the wound was created on the cells by sterile pipette tip. After that, the cells were rinsed with PBS to remove debris and incubated again in a 5% CO_2_ incubator at 37°C. The scratched wounds were viewed and photographed at 0 and 48 h, and ImageJ software was used to calculate the scratch area.

### Immunofluorescence Staining

After fixation with 4% paraformaldehyde for 10 min, the BMFs were permeated with 0.5% Triton X-100 (Solarbio; Beijing, China) for 2 min, and blocked with 3% BSA (BioFRo; Einhausen, Germany) for 0.5 h. Next, the cells were left to react with the primary antibodies against collagen I (1:200, Abcam; Shanghai, China) and α-SMA (1:200, Abcam) overnight for 12 h at 4°C. Afterwards, the cells were left to react with the second goat anti-rabbit antibodies (1:500, Abcam) at room temperature and counterstained with DAPI (Beyotime, Shanghai, China). The results were examined under a microscope (Ts2-FC, Nikon; Tokyo, Japan) at 200X magnification and representative fields were selected for analysis.

### Western Blot Analysis

The total proteins were isolated from cells by lysis buffer (RIPA, Beyotime). Then the concentration of the protein was assessed with BCA protein assay kit (Solarbio). The proteins were resolved via SDS-PAGE gels and transferred onto PVDF membranes (GE Healthcare Life; Munich, Germany). After being blocked with 5% skimmed milk for 2 h, the PVDF membranes were left to react at 4°C overnight with primary antibodies against Smad2 (1:1000, Afinity; Cincinnati, OH, USA), p-Smad2 (1:1000, Afinity), Smad3 (1:1000, Afinity), p-Smad3 (1:1000, Afinity), TGF-β1 (1:1000, Afinity), collagen I (1:1000, Afinity), α-SMA (1:1000, Afinity) and GAPDH (1:5000, Afinity). Subsequently, the membranes were rinsed and left to react with HRP-conjugated secondary antibodies at 37°C for 1 h. The protein bands were assessed using ECL reagent. The relative protein expression was quantified using Image J.

### Statistical Analysis

All of the trials were performed in triplicate on three separate occasions. The data were presented as mean ± SD, and analysed using SPSS 16.0 (IBM; Armonk, NY, USA). One-way ANOVA and SNK tests were applied for multigroup comparison. The Kruskal-Wallis H-test was applied if variances were not equal. p < 0.05 was considered statistically significant.

## Results

The data set used and analysed in this study can be obtained from the corresponding author upon reasonable request.

### AR Increased miR-497 mRNA Expression in BMFs

**Fig 1:**
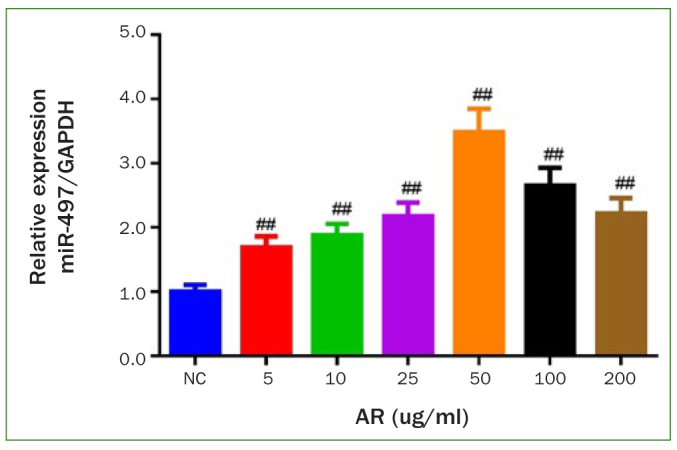
miR-497 mRNA expression level is increased in AR-stimulated buccal mucosal fibroblasts. The expression level of miR-497 mRNA was detected by qRT-PCR. #p < 0.05 and ##p < 0.01 vs NC. Data are presented as mean ± SD. n = 3. AR: arecoline; NC: normal control.

The effect of AR on the expression of miR-497 mRNA in BMFs at different concentrations (5–200 µg/ml) was examined to determine the suitable concentration of AR for in-vitro experiments. The results (Fig 1) showed obviously increased miR-497 mRNA expression at all doses of AR groups (p < 0.01). Moreover, the miR-497 mRNA expression level reached a maximum at 50 µg/ml. Therefore, the concentration of 50 µg/ml was chosen for the follow-up experiments.

### miR-497 Inhibition Decreased the Contractile Capacity of AR-induced BMFs

**Fig 2:**
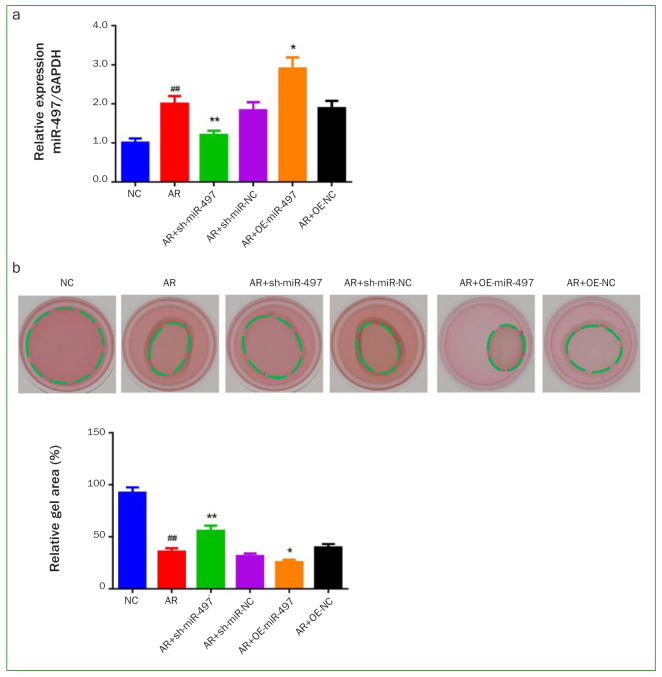
Inhibition of miR-497 decreases the contractile capacity of AR-induced BMFs (buccal mucosal fibroblasts). (a) miR-497 mRNA expression in AR-induced BMFs was inhibited or overexpressed after transfection, miR-497 mRNA expression was detected by qRT-PCR. (b) The contractile capacity of BMFs was assessed by collagen gel contraction assay. #p < 0.05 and ##p < 0.01 vs NC. *p < 0.05 and **p < 0.01 vs AR. Data are presented as mean ± SD. n = 3. NC: normal control; AR: arecoline; OE: overexpression; BMFs: buccal mucosal fibroblasts.

After cell transfection, the inhibited or overexpressed expression of miR-497 in AR-stimulated BMFs were confirmed by qRT-PCR results (Fig 2a). After that, the contractile capacity of the cells was examined. The collagen gel contraction assay showed that the cells in the AR group had an enhanced contractile capacity compared to the NC group (p < 0.01, Fig 2b). In contrast, the increased contractile capacity induced by AR decreased statistically significantly by inhibiting miR-497 (p < 0.01), while miR-497 overexpression further enhanced the contractile capacity (p < 0.05).

### Knockdown of miR-497 Prevented AR-induced BMFs Migration and Invasion Abilities

**Fig 3:**
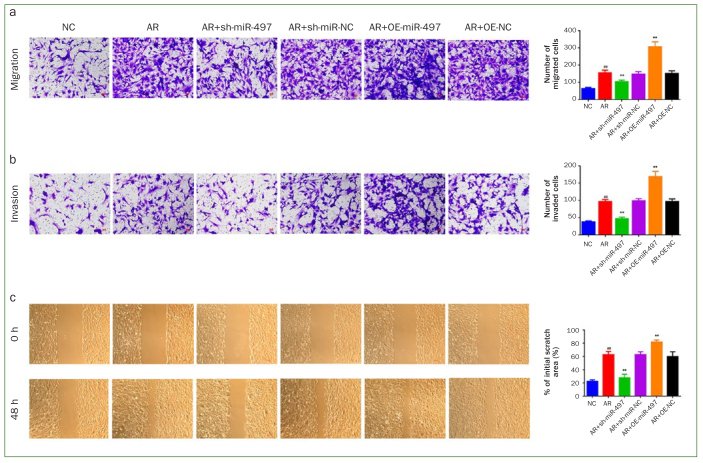
Silence of miR-497 diminishes the migration and invasion abilities. (a) The migration capacity and (b) invasion capacity of BMFs were measured by Transwell assay (200X). (c) The migration capacity of BMFs was assessed by wound healing assay (40X). #p < 0.05 and ##p < 0.01 vs NC. *p < 0.05 and **p < 0.01 vs AR. Data are presented as mean ± SD. n = 3. NC: normal control; AR: arecoline; OE: overexpression; BMFs: buccal mucosal fibroblasts.

The migration and invasion abilities of the BMFs were assessed with the Transwell and wound healing assays to explore whether miR-497 participated in the myofibroblast transdifferentiation of BMFs. The Transwell assay revealed that relative to the NC group, the migration and invasion abilities of the BMFs were dramatically enhanced in groups AR, AR+sh-miR-NC and AR+OE-NC (p < 0.01). More importantly, the increased migration and invasion abilities stimulated by AR were effectively prevented by miR-497 inhibition (p < 0.01), but further enhanced by miR-497 overexpression (p < 0.01, Figs 3a and 3b). The wound healing assay yielded results similar to those of the Transwell assay (Fig 3c).

### Inhibition of miR-497 Blunted Collagen I and α-SMA Expression in AR-induced BMFs

**Fig 4:**
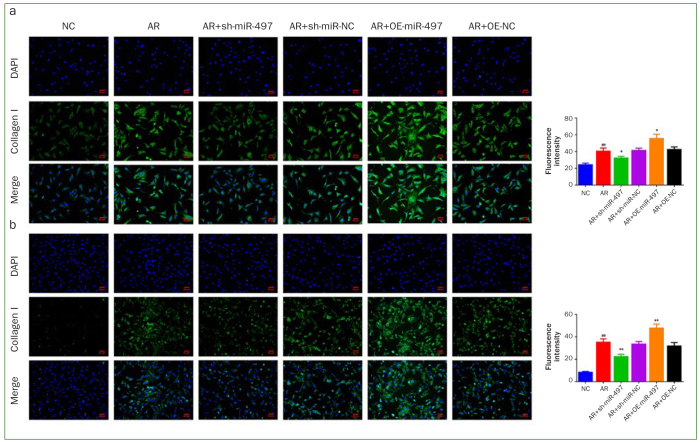
Knockdown of miR-497 blunts the expression of fibrosis-associated markers. (a) The expression of collagen I and (b) α-SMA was detected by immunofluorescence staining (200X). #p < 0.05 and ##p < 0.01 vs NC. *p < 0.05 and **p < 0.01 vs AR. Data are presented as mean ± SD. n = 3. NC: normal control; AR: arecoline; OE: overexpression; BMFs: buccal mucosal fibroblasts.

**Fig 5:**
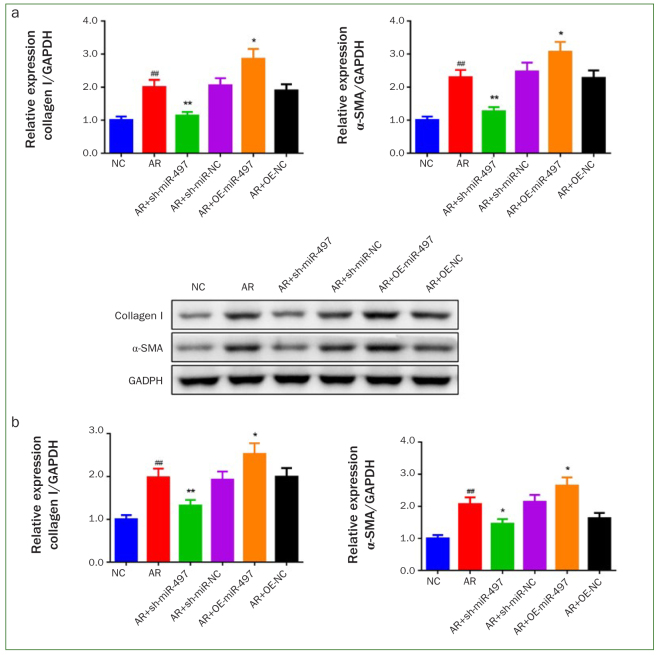
Inhibition of miR-497 decreases the mRNA and protein expression of collagen I, α-SMA. (a) The mRNA expression of collagen I and α-SMA were measured by qRT-PCR. (b) The protein expression of collagen I and α-SMA was assessed by western blot. #p < 0.05 and ##p < 0.01 vs NC. *p < 0.05 and **p < 0.01 vs AR. Data are presented as mean ± SD. n = 3. NC: normal control; AR: arecoline; OE: overexpression.

Collagen I and α-SMA expression level were measured to further explore the effect of miR-497 inhibition on the myofibroblast transdifferentiation in BMFs. As presented in Fig 4, the results of immunofluorescence staining suggested that the expression level of collagen I and α-SMA were dramatically upregulated in AR, AR+sh-miR-NC, AR+OE-NC groups (p < 0.01). Nevertheless, suppression of miR-497 remarkably blunted collagen I and α-SMA expression (p < 0.05), and overexpression of miR-497 evidently promoted the expression level of collagen I and α-SMA in AR-induced BMFs (p < 0.05). The findings of qRT-PCR and western blot were in line with these results (Fig 5).

### Knockdown of miR-497-mediated Myofibroblast Transdifferentiation in BMFs through TGF-β1/Smads Signaling Pathway

**Fig 6:**
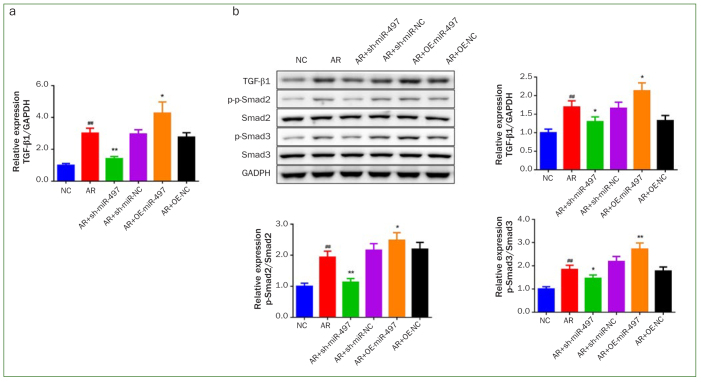
Knockdown of miR-497 mediated myofibroblast transdifferentiation in BMFs through TGF-β1/Smads signaling pathway. (A) The mRNA expression of TGF-β1 was measured by qRT-PCR. (B) The TGF-β1 protein expression, Smad2 and Smad3 phosphorylation were measured by western blot. #p < 0.05 and ##p < 0.01 vs NC. *p < 0.05 and **p < 0.01 vs AR. Data are presented as mean ± SD. n = 3. NC: normal control, AR: arecoline; OE: overexpression.

To further investigate the molecular mechanism of miR-497 mediated myofibroblast transdifferentiation in BMFs, the gene and protein expression related to TGF-β1/Smads signaling pathway were measured. The results of qRT-PCR as well as western blotting indicated that AR statistically significantly upregulated TGF-β1 expression level and the phosphorylation of its downstream proteins Smad2 and Smad3 (p < 0.05, Fig 6). However, miR-497 silencing statistically significantly decreased the expression of TGF-β1, phosphorylation of Smad2 and Smad3 (p < 0.05), while miR-497 overexpression statistically significantly increased these (p < 0.05).

## Discussion

OSF is a chronic fibrogenic disorder marked by the formation of fibrotic band and atrophic epithelium, eventually causing restricted mouth opening.^31^ There are some oral habits associated with the risk of OSF, such as alcohol drinking, cigarette smoking etc, but betel-nut chewing is the main risk factor.^33,36^ Studies have confirmed that the initial risk factor for OSF in betel-nut chewers is linked to AR exposure.^1^ Betel-nut mixture contacts with the oral tissues are cumulative, resulting in constant stimulation of the oral tissues by the AR in the betel nut.^18^ AR promotes the myofibroblast transdifferentiation in BMFs, which promotes the progression of OSF.^4,27^ Thus, AR-stimulated BMFs were employed as a cellular model of OSF in the study.^7,23^

miR-497, as one of the members of miR-15/16/195/ 424/497 family, is located on chromosome 17p13.124. Although there is no evidence that miR-497 contributes to the development of OSF, some studies have suggested that miR-497 can enhance the TGF-β/Smad pathway to promote hepatic fibrosis by targeting Smad7 both in-vivo and in-vitro.^39^ Furthermore, miR-497 inhibition was found to attenuate silica-induced pulmonary fibrosis by targeting Smad3 and Bcl-220. It is known that Smad as well as Bcl-2 are dysregulated in OSF patients.^22^ To fully understand the effect of miR-497 on OSF, miR-497 was silenced or overexpressed in this study. Subsequently, miR-497 inhibition was observed to attenuate the development of OSF by inhibiting myofibroblast transdifferentiation in BMFs.

After injury, fibroblasts are activated, migrate to the wound and differentiate into myofibroblasts for wound contraction and healing.^30^ Hence, collagen gel contraction, migration and invasion abilities can be applied to assess the myofibroblast activity in general. It has been revealed that collagen contractility is enhanced in a dose-dependent manner following treatment with betel-nut extract.^3^ Liao et al^21^ also revealed that BMFs show greater contractility, enhanced migration and invasion abilities under AR stimulation. Consistent with previous findings, this study found that AR statistically significantly upregulated the contractility, migration and invasion abilities of BMFs, while inhibition of miR-497 counteracted this. Thus, our results indicated that suppression of miR-497 could inhibit AR-induced myofibroblast transdifferentiation in BMFs.

α-SMA and collagen I are common biomarkers of fibrosis.^8^ Increased α-SMA and collagen I expression provoke the transdifferentiation of fibroblasts into myofibroblasts.^26^ Research has shown that modulating α-SMA and collagen I expression is an efficient approach to treating or preventing fibrosis. Meng et al^25^ have reported that Bekhogainsam decoction is effective for treating renal fibrosis in diabetic nephropathy, based on its effect on the reduction of PKCα/TGF-β1/α-SMA expression. Some studies have reported that the TGF-β1 signaling pathway is a target in treating irradiation-induced organ fibrosis for decreasing collagen I and II expression level.^6,11^ Furthermore, the examination of 35 OSF specimens showed that 80% of cases presented strong expression of α-SMA, relative to normal mucosal tissues.^37^ In the present study, the mRNA and protein expression of α-SMA and collagen I were notably blunted in AR-exposed BMFs after miR-497 inhibition. Combining the above studies and the findings of the current study, it is hypothesised that miR-497 silencing might inhibit AR-induced myofibroblast transdifferentiation by modulating α-SMA and collagen I.

To obtain a more detailed understanding of the molecular mechanism of miR-497 in OSF, the effect of miR-497 on the TGF-β1/Smads signaling pathway was investigated. TGF-β1 is primarily responsible for the deposition of extracellular matrix protein.^14^ As an essential profibrotic factor, TGF-β1 is always overexpressed in the tissues of patients with fibrotic diseases.^19^ Research has shown that the development of OSF is due to the activation of the TGF-β pathway.^15^ Smad2 and Smad3 are downstream proteins of the TGF-β1 signaling pathway, and the upregulation of Smad2 and Smad3 expression are regulated by TGF-β1 activation.^10^ Smad proteins play different roles in fibrosis; some Smads are profibrosis (Smad2 and Smad3), whereas others are antifibrosis (Smad6 and Smad7).^16^ A study conducted by Ku et al^17^ observed that the expression levels of TGF-β and p-Smad2/3 protein were clearly elevated in the AR-induced rat model of cardiac fibrosis. Notably, other studies have demonstrated that inhibition of miR-497 can effectively suppress the TGF-β1/Smads signaling pathway.^2,5^ Consistent with previous studies, this research discovered that inhibition of miR-497 greatly weakened TGF-β1 expression as well as Smad2 and Smad3 phosphorylation, suggesting that the protective effect of miR-497 on AR-induced myofibroblast transdifferentiation may mediate via the TGF-β1/Smads signaling pathway.

Nevertheless, this study has a few limitations. First, only one cell line was used in this study, which may not be broadly representative. Second, this study explored the effect and mechanism of miR-497 in a cellular model, but lacked corresponding in-vivo experiments to validate the effect of miR-497 on OSF. In the future, additional cell lines and animal models will be used to better demonstrate the effect and mechanism of miR-497 on OSF.

## Conclusion

This study demonstrated that miR-497 inhibition may exert a protective effect in AR-induced OSF in-vitro via the inhibition of myofibroblast transdifferentiation by targeting the TGF-β1/Smads signaling pathway. These results indicate that miR-497 may serve as a screening factor and potential therapeutic target for OSF.
